# A novel method of constructing compactly supported orthogonal scaling functions from splines

**DOI:** 10.1186/s13660-017-1425-9

**Published:** 2017-06-29

**Authors:** Shouzhi Yang, Huiqing Huang

**Affiliations:** 1 Department of Mathematics, Shantou University, Shantou, Guangdong 515063 P.R. China; 2 School of Mathematics, Jiaying University, Meizhou, Guangdong 514015 P.R. China

**Keywords:** B-spline, orthogonal, compactly supported, scaling, MRA

## Abstract

A novel construction of compactly supported orthogonal scaling functions and wavelets with spline functions is presented in this paper. Let $M_{n}$ be the center B-spline of order *n*, except for the case of order one, we know $M_{n}$ is not orthogonal. But by the formula of orthonormalization procedure, we can construct an orthogonal scaling function corresponding to $M_{n}$. However, unlike $M_{n}$ itself, this scaling function no longer has compact support. To induce the orthogonality while keeping the compact support of $M_{n}$, we put forward a simple, yet efficient construction method that uses the formula of orthonormalization procedure and the weighted average method to construct the two-scale symbol of some compactly supported orthogonal scaling functions.

## Introduction

It is well known that B-splines have many useful properties, and they are widely used in practical problems. But except for the case of order one, B-splines of other orders are not orthogonal [1]. Thus, in order to get the property of orthogonality, many researchers are interested in the study of constructing orthogonal wavelets with B-splines [2–6]. For instance, Franklin wavelet and Battle-Lemarié wavelets [7, 8], but these wavelets are not compactly supported. In [5] Goodman gave a construction, for any $n\geq 2$, of a space *S* of spline functions of degree $n-1$ with simple knots in $\frac{1}{4}\mathbb{Z}$ which is generated by a triple of refinable, orthogonal functions with compact support. Subsequently, Cho and Lai simplified Goodman’s constructive steps for compactly supported orthonormal scaling functions and provided an inductive method for constructing compactly supported orthonormal wavelets [6]. In [2] Nguyen and He presented a method to construct orthogonal spline-type scaling functions with B-splines. They multiplied a class of polynomial function factors to the two-scale symbol of the B-splines so that they become the two-scale symbol of a spline-type orthogonal compactly supported function. Different from above, firstly, we use orthonormalization procedure so that splines become orthogonal scaling functions. Unfortunately, the orthogonal scaling functions are not compactly supported. So, in order to make them have the property of compact support, we use the weighted average method to eliminate the denominator of the two-scale symbol, which is the corresponding orthogonal scaling function. And from examples in Section 3, we found that this method is simple and flexible.

The goal of this section is to prepare for the next chapter of theorem proving. For this reason, we need the following auxiliary results.

### Definition 1.1

[9]

*A Multiresolution Analysis* (*MRA*) comprises a sequence of closed subspaces $V_{j} $, $j\in \mathbb{Z}$, of $L_{2}(\mathbb{R})$ satisfying

(i) (Nested) $V_{j} \subset V_{j+1}$ for all $j\in \mathbb{Z}$;
(ii) (Density) $\overline{\bigcup_{j\in \mathbb{Z}}V_{j}}=L_{2}( \mathbb{R})$;
(iii) (Separation) $\overline{\bigcap_{j\in \mathbb{Z}}V_{j}}=\{0\}$;
(iv) (Scaling) $f(x) \in V_{j}$ if and only if $f(2x) \in V_{j+1}$ for all $j\in \mathbb{Z}$;
(v) (Basis) There exists a function $\phi \in V_{0}$ such that $\{ \phi (x-k): k\in \mathbb{Z}\}$ is an orthonormal basis or a Riesz basis for $V_{0}$.

The function *ϕ* defined as in Definition 1.1 is called the scaling function of the given MRA. From (iv), we know that $\phi \in V_{0}$ is also in $V_{1}$. Since $\{\phi_{1,k}:=2^{1/2} \phi (2x-k):k\in \mathbb{Z}\}$ is a Riesz basis of $V_{1}$, then there exists a unique $l^{2}$-sequence $\{p_{k}\}$ satisfying the ‘two-scale relation’

1.1 $$ \phi (x)=\sum_{k=-\infty }^{\infty } p_{k}\phi (2x-k). $$

This sequence $\{p_{k}\}$ is called the ‘two-scale sequence’ of *ϕ*. With this $l^{2}$-sequence, we define

1.2 $$ P(\omega )=\frac{1}{2}\sum_{k=-\infty }^{\infty } p_{k}e^{-i\omega k}. $$

Then the Fourier transform formulation of identity (1.1) can be written as

1.3 $$ \widehat{\phi }(\omega )=P \biggl(\frac{\omega }{2} \biggr) \widehat{\phi } \biggl( \frac{ \omega }{2} \biggr). $$

We call $P(\omega )$ the two-scale symbol of the scaling function *ϕ*. Noticing that $\{\phi (x-k): k\in \mathbb{Z} \}$ is an orthonormal basis, we have the following equivalent statements of orthogonality, see also in [6, 9–12].

### Theorem 1.2

*Suppose that* $P(\omega )=\frac{1}{2}\sum_{k}p _{k}e^{-i\omega k}$ *is a polynomial satisfying the following conditions*:

1.4 $$\begin{aligned}& P(0)=1, \end{aligned}$$

1.5 $$\begin{aligned}& \bigl\vert P(\omega )\bigr\vert ^{2}+\bigl\vert P(\omega +\pi ) \bigr\vert ^{2}=1, \end{aligned}$$

1.6 $$\begin{aligned}& \bigl\vert P(\omega )\bigr\vert >0,\quad \forall -\pi /2< \omega < \pi /2. \end{aligned}$$

*Then* $P(\omega )$ *is the two*-*scale symbol of an orthogonal scaling function*.

### Riesz lemma

*Let* $a_{0},\ldots,a_{N}$ *be real numbers and* $a_{N}\neq 0$ *such that*

1.7 $$ A(\omega ):=\frac{a_{0}}{2}+\sum_{k=1}^{N} a_{k} \cos (k\omega ) \geq 0, \quad \forall \omega \in \mathbb{R}. $$

*Then there exists a polynomial*

1.8 $$ B(z)=\sum_{k=0}^{N}b_{k}z^{k} $$

*with real coefficients and exact degree* *N* *satisfying*

1.9 $$ \bigl\vert B(z)\bigr\vert ^{2}=A(\omega ),\quad z=e^{-i\omega } . $$

## Constructing compactly supported orthogonal scaling functions

In this section we will give a new method to construct compactly supported orthogonal scaling functions and wavelets by a center cardinal B-spline. The *m*th order center cardinal B-spline $M_{m}$ is defined as follows, see also [9, 13].

2.1 $$ M_{m}(x)=\frac{1}{(m-1)!}\sum _{k=0}^{m}(-1)^{k} \left ( \begin{matrix}m \\ k \end{matrix} \right ) \biggl(x+ \frac{m}{2}-k \biggr)_{+}^{m-1}. $$

It is well known that $M_{m}(x)$ is symmetric with respect to the origin and $\operatorname{supp}M_{m}=[-m/2,m/2]$.

Denote

2.2 $$ \Omega_{m}(\omega )=\sum_{k\in \mathbb{Z}} \bigl\vert \widehat{M}_{m}(\omega +2k \pi )\bigr\vert ^{2}. $$

Then, for any $m\geq 2$, there exists a positive constant $A_{m}$ such that

2.3 $$ A_{m}\leq \Omega_{m}(\omega )\leq 1,\quad \forall \omega \in \mathbb{R}. $$

Furthermore, it is easy to verify that

2.4 $$ \Omega_{m}(\omega )=1-4\sum _{k=1}^{m-1}M_{2m}(k) \sin ^{2} \biggl(\frac{k\omega }{2} \biggr). $$

Note that the scaling function $M_{m}(x)$ is semi-orthogonal for $m \geq 2$. Next, we give a method to obtain the orthogonal scaling function through the B-spline $M_{m}$. We define a function $\varphi_{m}(x)$ through its Fourier transform

2.5 $$ \widehat{\varphi }_{m}(\omega )=\frac{\widehat{M}_{m}(\omega )}{ ( \sum_{k\in \mathbb{Z}}\vert \widehat{M}_{m}(\omega +2k\pi )\vert ^{2} ) ^{1/2}}. $$

Since $\Omega_{m}(\omega )=\sum_{k\in \mathbb{Z}}\vert \widehat{M}_{m}(\omega +2k\pi )\vert ^{2}$, then

2.6 $$ \widehat{\varphi }_{m}(\omega )=\Omega_{m}( \omega )^{-1/2}\widehat{M} _{m}(\omega ). $$

By (1.3) and (2.6), we can obtain $P_{m}(\omega )$,

2.7 $$ P_{m}(\omega )=\frac{\widehat{\varphi }_{m}(2\omega )}{ \widehat{\varphi }_{m}(\omega )}= \biggl( \frac{\Omega_{m}(\omega ) }{ \Omega_{m}(2\omega )} \biggr) ^{1/2}\cos^{m}(\omega /2), $$

which is the two-scale symbol of the scaling function $\varphi_{m}(x)$. It is well known that the scaling function $\varphi_{m}(x)$ determined by (2.7) is orthogonal but not compactly supported. So next we concentrate our effort on the study of constructing compactly supported orthogonal scaling functions.

Note that the presence of the denominator in (2.7) can bring about scaling functions which are not compactly supported. Therefore, we multiply a function factor to the two-scale symbol $P_{m}(\omega )$ and obtain the following Theorem 2.1 and some corollaries.

### Theorem 2.1

*For* $i=1,\ldots ,N$, *suppose that* $h_{i}( \omega )$ *is the two*-*scale symbol of an orthogonal scaling function*, *and let*

2.8 $$ \bigl\vert H(\omega ) \bigr\vert ^{2}=\sum _{i=1}^{N}\lambda_{i}(\omega ) \bigl\vert h _{i}(\omega ) \bigr\vert ^{2}, $$

*where* $\lambda_{i}(\omega )$ *is a* *π*-*periodic function and satisfies the following conditions*:

$$\textstyle\begin{cases} 0\leq \lambda_{i}(\omega )\leq 1, \\ \sum_{i=1}^{N}\lambda_{i}(\omega )=1. \end{cases} $$

*Then* $H(\omega )$ *is the two*-*scale symbol of an orthogonal scaling function*.

### Proof

It is easy to observe that $H(\omega )$ satisfies statements (1.4) and (1.6) in Theorem 1.2, now we only need to prove that $H(\omega )$ also satisfies $\vert H(\omega ) \vert ^{2}+ \vert H( \omega +\pi ) \vert ^{2}=1$. Since $h_{i}(\omega )$ is the two-scale symbol of an orthogonal scaling function, we have

$$\bigl\vert h_{i}(\omega ) \bigr\vert ^{2}+ \bigl\vert h_{i}(\omega +\pi ) \bigr\vert ^{2}=1,\quad \mbox{for } i=1, \ldots ,N. $$

Thus

$$\begin{aligned} \bigl\vert H(\omega ) \bigr\vert ^{2}+ \bigl\vert H(\omega +\pi ) \bigr\vert ^{2} &=\sum_{i=1}^{N} \lambda_{i}(\omega ) \bigl\vert h_{i}(\omega ) \bigr\vert ^{2}+\sum_{i=1}^{N} \lambda_{i}(\omega +\pi ) \bigl\vert h_{i}(\omega +\pi ) \bigr\vert ^{2} \\ &=\sum_{i=1}^{N}\lambda_{i}( \omega ) \bigl\vert h_{i}(\omega ) \bigr\vert ^{2}+ \sum _{i=1}^{N}\lambda_{i}(\omega ) \bigl\vert h_{i}(\omega +\pi ) \bigr\vert ^{2} \\ &=\sum_{i=1}^{N}\lambda_{i}( \omega )=1, \end{aligned}$$

which by Theorem 1.2 implies that $H(\omega )$ is the two-scale symbol of an orthogonal scaling function. □

### Corollary 2.2

*Let* $m\geq 2$ *be any integer*, $\lambda_{1}( \omega )=\Omega_{m}(2\omega )$, $\lambda_{2}(\omega )=1-\Omega_{m}(2 \omega )$, $P_{m}(\omega )$ *be defined as in* (2.7). *Suppose*

2.9 $$\begin{aligned} \bigl\vert \widetilde{P}_{m}(\omega )\bigr\vert ^{2} =& \Biggl( 1-4\sum_{k=1}^{m-1}M_{2m}(k) \sin^{2} \biggl(\frac{k\omega }{2} \biggr) \Biggr) \cos^{2m}( \omega /2) \\ &{}+ 2\sum_{k=1} ^{m-1}M_{2m}(k) \sin^{2}(k\omega ) \end{aligned}$$

*and*

2.10 $$ \bigl\vert H_{m}(\omega )\bigr\vert ^{2}= \lambda_{1}(\omega )\bigl\vert P_{m}(\omega )\bigr\vert ^{2}+\lambda _{2}(\omega )\bigl\vert \widetilde{P}_{m}( \omega )\bigr\vert ^{2}. $$

*Then* $H_{m}(\omega )$ *is a two*-*scale symbol of some compactly supported orthogonal scaling function*.

### Proof

By (2.4) and (2.7), we have

2.11 $$ P_{m}(\omega )= \biggl( \frac{1-4\sum_{k=1}^{m-1}M_{2m}(k)\sin ^{2}(\frac{k \omega }{2})}{1-4\sum_{k=1}^{m-1}M_{2m}(k)\sin^{2}(k\omega )} \biggr) ^{1/2} \cos^{m}(\omega /2), $$

therefore

2.12 $$ \bigl\vert P_{m}(\omega )\bigr\vert ^{2}= \biggl( \frac{1-4\sum_{k=1}^{m-1}M_{2m}(k)\sin^{2}(\frac{k \omega }{2})}{1-4 \sum_{k=1}^{m-1}M_{2m}(k)\sin^{2}(k\omega )} \biggr) \cos^{2m}(\omega /2). $$

Since $P_{m}(\omega )$ is a two-scale symbol of some orthogonal scaling function, we obtain

$$\begin{aligned} 1 =&\bigl\vert P_{m}(\omega )\bigr\vert ^{2}+\bigl\vert P_{m}(\omega +\pi)\bigr\vert ^{2} \\ =& \Biggl( \Biggl( 1-4\sum_{k=1}^{m-1}M_{2m}(k) \sin^{2} \biggl( \frac{k\omega }{2} \biggr) \Biggr) \cos^{2m} \biggl(\frac{\omega }{2} \biggr) \\ &{}+ \Biggl( 1-4\sum_{k=1}^{m-1}M_{2m}(k) \sin^{2} \biggl(\frac{k(\omega +\pi )}{2} \biggr) \Biggr) \sin ^{2m} \biggl(\frac{\omega }{2} \biggr) \Biggr) \\ &{} \Big/ \Biggl(1-4\sum_{k=1}^{m-1}M_{2m}(k) \sin ^{2}(k\omega ) \Biggr). \end{aligned}$$

Multiplying $1-4\sum_{k=1}^{m-1}M_{2m}(k)\sin ^{2}(k\omega )$ on both sides in the above equation, we obtain

$$\begin{aligned}& 1-4\sum_{k=1}^{m-1}M_{2m}(k)\sin ^{2}(k \omega ) \\& \quad = \Biggl( 1-4\sum_{k=1}^{m-1}M_{2m}(k) \sin ^{2} \frac{k\omega }{2} \Biggr) \cos^{2m} \frac{\omega }{2} \\& \qquad {}+ \Biggl( 1-4\sum_{k=1}^{m-1}M_{2m}(k) \sin ^{2} \frac{k( \omega +\pi )}{2} \Biggr) \sin^{2m} \frac{\omega }{2}, \end{aligned}$$

it means that

2.13 $$\begin{aligned} 1 ={}& \Biggl( 1-4\sum_{k=1}^{m-1}M_{2m}(k) \sin ^{2} \frac{k\omega }{2} \Biggr) \cos^{2m} \frac{\omega }{2}+ \Biggl( 1-4\sum_{k=1}^{m-1}M_{2m}(k) \sin ^{2} \frac{k( \omega +\pi )}{2} \Biggr) \sin^{2m} \frac{\omega }{2} \\ &{}+4\sum_{k=1}^{m-1}M_{2m}(k)\sin ^{2}(k \omega ). \end{aligned}$$

Therefore

2.14 $$ \bigl\vert \widetilde{P}_{m}(\omega )\bigr\vert ^{2}+\bigl\vert \widetilde{P}_{m}(\omega +\pi )\bigr\vert ^{2}=1. $$

By Theorem 2.1 and the Riesz lemma, we know that $H_{m}(\omega )$ is a two-scale symbol of some compactly supported orthogonal scaling function. □

### Corollary 2.3

*Let* $m_{1},\ldots ,m_{N}\geq 2$ *be any integer*. *Define* $h_{i}(\omega )=P_{m_{i}}(\omega )$ *as in* (2.7) *and* $\vert \widetilde{P}_{m_{i}}(\omega )\vert ^{2}$ *as in* (2.9) *for* $i=1,\ldots , N$. *Assume that*

$$h_{N+1}(\omega )=\sqrt{ \Biggl(\sum _{i=1}^{N}\bigl\vert \widetilde{P}_{m_{i}}( \omega)\bigr\vert ^{2} \Biggr)\Big/N} $$

*and*

2.15 $$ \bigl\vert h(\omega )\bigr\vert ^{2}=\sum _{i=1}^{N+1} \lambda_{i}(\omega )\bigl\vert h_{i}(\omega )\bigr\vert ^{2}, $$

*where* $\lambda_{i}(\omega )$ *satisfies the following conditions*:

$$\textstyle\begin{cases} \lambda_{i}(\omega )=a_{i}g(\omega )\quad (i=1,\ldots ,N), \\ \sum_{i=1}^{N} a_{i}(\omega )=1,\quad 0\leq a_{i} \leq 1, \\ g(\omega )=\Omega_{m_{1}}(2\omega )\times \Omega_{m_{2}}(2\omega ) \times \cdots \times \Omega_{m_{N}}(2\omega ), \\ \lambda_{N+1}(\omega )=1-g(\omega ). \end{cases} $$

*Then* $h(\omega )$ *is the two*-*scale symbol of a compactly supported orthogonal scaling function*.

The proof is analogous to that of Corollary 2.2.

### Corollary 2.4

*Define*

$$\lambda_{1}(\omega )= \bigl(a+b\sin^{2}\omega +c \sin^{2}(2\omega ) \bigr) \biggl(1- \frac{2}{3}\sin^{2} \omega \biggr) $$

*and*

$$\lambda_{2}(\omega )= \bigl(d+e\sin^{2}\omega \bigr) \biggl(1-\frac{13}{15}\sin^{2} \omega -\frac{1}{30} \sin^{2}(2\omega ) \biggr), $$

*where the real numbers* *a*, *b*, *c*, *d*, *e* *satisfy*

$$0\leq a \leq 26,\qquad b=\frac{21}{5}-\frac{13}{15}a,\qquad c= \frac{1}{5}- \frac{a}{30},\qquad d=1-a, \qquad e= \frac{2}{3}a-4. $$

*Moreover*, *let*

2.16 $$ \bigl\vert P(\omega )\bigr\vert ^{2}= \lambda_{1}(\omega )\bigl\vert P_{2}(\omega )\bigr\vert ^{2}+\lambda_{2}( \omega )\bigl\vert P_{3}( \omega )\bigr\vert ^{2}, $$

*where* $P_{2}(\omega )$ *and* $P_{3}(\omega )$ *are defined as in* (2.7). *Then* $P(\omega )$ *is the two*-*scale symbol of a compactly supported orthogonal scaling function*.

To facilitate our proof of Corollary 2.4, we need the following result.

### Lemma 2.5

*Define*

$$\lambda_{1}(\omega )= \bigl(a+b\sin^{2}\omega +c \sin^{2}(2\omega ) \bigr) \biggl(1- \frac{2}{3}\sin^{2} \omega \biggr) $$

*and*

$$\lambda_{2}(\omega )= \bigl(d+e\sin^{2}\omega \bigr) \biggl(1-\frac{13}{15}\sin^{2} \omega -\frac{1}{30} \sin^{2}(2\omega ) \biggr), $$

*where* *a*, *b*, *c*, *d* *and* *e* *are real numbers*. *Then there exist real numbers* *a*, *b*, *c*, *d* *and* *e* *satisfying*

2.17 $$\begin{aligned} \lambda_{1}(\omega )+\lambda_{2}(\omega )=1. \end{aligned}$$

### Proof

By (2.17) we obtain

$$\begin{aligned} 1={} &a+d+ \biggl\{ b-\frac{2}{3}a-\frac{13}{15}d+e- \biggl[ \biggl( \frac{4}{30}d-4c \biggr)\cos^{2} \omega + \biggl( \frac{2}{3}b+\frac{13}{15}e \biggr)\sin^{2}\omega \biggr] \\ &{}- \biggl(\frac{2}{3}c+\frac{e}{30} \biggr)\sin^{2}2 \omega \biggr\} \sin^{2}\omega . \end{aligned}$$

Now, consider the system

2.18 $$ \textstyle\begin{cases} a+d=1, \\ b-\frac{2}{3}a-\frac{13}{15}d+e=\frac{2}{3}b+\frac{13}{15}e, \\ \frac{2}{3}b+\frac{13}{15}e=\frac{4}{30}d-4c, \\ \frac{2}{3}c+\frac{e}{30}=0. \end{cases} $$

It is easy to check that the pair number $(a,b,c,d,e)$ satisfying

$$b=\frac{21}{5}-\frac{13}{15}a,\qquad c=\frac{1}{5}- \frac{a}{30},\qquad d=1-a,\qquad e= \frac{2}{3}a-4 $$

is the solution of (2.18), also the solution of (2.17). □

### Lemma 2.6

*Let* $0 \leq x \leq 1$ *and* $0 \leq a \leq 26$. *Then*

2.19 $$\begin{aligned}& 1+2x+ \biggl(\frac{47}{5}-\frac{7a}{5} \biggr)x^{2}+ \biggl( \frac{182a}{45}-\frac{394}{15} \biggr)x ^{3}+ \biggl(16- \frac{8a}{3} \biggr)x^{4}+ \biggl( \frac{16a}{45}- \frac{32}{15} \biggr)x^{5} \\& \quad >0. \end{aligned}$$

### Proof

Define

2.20 $$\begin{aligned} f(a)={}&1+2x+\frac{47}{5}x^{2}- \frac{394}{15}x^{3}+16x^{4}- \frac{32}{15}x ^{5} \\ &{}+ \biggl(-\frac{7}{5}x^{2}+ \frac{182}{45}x^{3}- \frac{8}{3}x^{4}+ \frac{16}{45}x^{5} \biggr)a, \end{aligned}$$

one obtains $f'(a)=-\frac{7}{5}x^{2}+\frac{182}{45}x^{3}-\frac{8}{3}x ^{4}+\frac{16}{45}x^{5}$, then

2.21 $$ \textstyle\begin{cases} f'(a)\leq 0, &x\in [0 , 0.5], \\ f'(a)\geq 0,& x\in [0.5 , 1]. \end{cases} $$

Now define

$$\textstyle\begin{cases} g_{1}(x)=1+2x+\frac{47}{5}x^{2}-\frac{394}{15}x^{3}+16x^{4}- \frac{32}{15}x^{5}, \\ g_{2}(x)=-\frac{7}{5}x^{2}+\frac{182}{45}x^{3}-\frac{8}{3}x^{4}+ \frac{16}{45}x^{5}, \end{cases} $$

then

2.22 $$ \textstyle\begin{cases} g_{1}'(x)=2+\frac{94}{5}x-\frac{394}{5}x^{2}+64x^{3}-\frac{32}{3}x ^{4}, \\ g_{2}'(x)=-\frac{14}{5}x+\frac{182}{15}x^{2}-\frac{32}{3}x^{3}+ \frac{16}{3}x^{4}. \end{cases} $$

Therefore

$$g_{1}(x)\geq 1,\qquad g_{2}(x)>-0.03746,\quad \forall x\in [0 , 0.5]. $$

This means that $f(a)>0.02604$ for all $x\in [0, 0.5]$ and $a\in [0, 26]$.

Similarly, one can obtain $f(a)>1.3322\times 10^{-15}$ for all $x\in [0.5 , 1]$ and $a\in [0 , 26]$. This completes the proof. □

### Proof of Corollary 2.4

By calculation, we have

$$\begin{aligned} \bigl\vert P(\omega )\bigr\vert ^{2}= {}& \biggl[1+2 \sin^{2}\frac{\omega }{2}+ \biggl(\frac{47}{5}- \frac{7a}{5} \biggr)\sin^{4} \frac{\omega }{2}+ \biggl( \frac{182a}{45}- \frac{394}{15} \biggr) \sin^{6} \frac{\omega }{2} \\ &{}+ \biggl(16-\frac{8a}{3} \biggr)\sin^{8}\frac{\omega }{2}+ \biggl(\frac{16a}{45}- \frac{32}{15} \biggr)\sin^{10} \frac{\omega }{2} \biggr]\cos^{4}\frac{\omega }{2}. \end{aligned}$$

Denote

$$\begin{aligned} A(\omega )= {}&1+2\sin^{2}\frac{\omega }{2}+ \biggl( \frac{47}{5}- \frac{7a}{5} \biggr) \sin^{4} \frac{\omega }{2}+ \biggl( \frac{182a}{45}-\frac{394}{15} \biggr) \sin^{6}\frac{ \omega }{2} \\ &{}+ \biggl(16-\frac{8a}{3} \biggr)\sin^{8}\frac{\omega }{2}+ \biggl(\frac{16a}{45}- \frac{32}{15} \biggr)\sin^{10} \frac{\omega }{2}, \end{aligned}$$

and note that $A(\omega )$ is an even and 2*π*-periodic function. We obtain from Lemma 2.6

$$A(\omega )>0,\quad \forall \omega \in \mathbb{R}. $$

Therefore, $\vert P(\omega )\vert ^{2}>0$.

Noticing that $P_{2}(\omega )$ and $P_{3}(\omega )$ are the two-scale symbols of orthogonal scaling functions and $\lambda_{1}(\omega )+ \lambda_{2}(\omega )=1$, we have

$$\begin{aligned} \bigl\vert P(\omega )\bigr\vert ^{2}+\bigl\vert P(\omega +\pi ) \bigr\vert ^{2}=1. \end{aligned}$$

Now applying Theorem 2.1 and the Riesz lemma, we know that $P(\omega )$ is a two-scale symbol of some compactly supported orthogonal scaling function. □

## Examples

In this section, we give three examples to show our construction scheme introduced in the above section.

### Example 3.1

For $m=3$, from (2.4) and (2.7), we have

$$\begin{aligned} \Omega_{3}(\omega )&=1-4\sum_{k=1}^{2}M_{6}(k) \sin^{2} \biggl( \frac{k\omega }{2} \biggr) \\ &=1-\frac{13}{15} \sin^{2}\frac{\omega }{2}- \frac{1}{30} \sin^{2}\omega \end{aligned}$$

and

3.1 $$\begin{aligned} \bigl\vert P_{3}(\omega )\bigr\vert ^{2}= \biggl( \frac{1-\frac{13}{15}\sin^{2}\frac{\omega }{2}-\frac{1}{30}\sin^{2}\omega }{1-\frac{13}{15}\sin^{2}\omega - \frac{1}{30}\sin^{2}(2\omega )} \biggr) \cos^{6}(\omega /2), \end{aligned}$$

respectively.

Moreover, we have

$$\begin{aligned} \bigl\vert \widetilde{P}_{3}(\omega )\bigr\vert ^{2}= \biggl(1- \frac{13}{15}\sin^{2}\frac{\omega }{2}- \frac{1}{30} \sin^{2}\omega \biggr)\cos^{6}(\omega /2)+ \frac{13}{30} \sin ^{2}\omega +\frac{1}{60} \sin^{2}(2\omega ). \end{aligned}$$

Now, we obtain from (2.10) that

$$\begin{aligned} \bigl\vert H_{3}(\omega )\bigr\vert ^{2} ={}& \biggl(1- \frac{13}{15} \sin^{2}\frac{\omega }{2}- \frac{1}{30} \sin^{2}\omega \biggr)\cos^{6}(\omega /2)+ \biggl( \frac{13}{15} \sin^{2}\omega +\frac{1}{30} \sin^{2}(2\omega ) \biggr) \\ &{}\times \biggl( \biggl(1-\frac{13}{15}\sin^{2} \frac{\omega }{2}- \frac{1}{30} \sin^{2}\omega \biggr) \cos^{6}(\omega /2)+ \frac{13}{30}\sin^{2}\omega + \frac{1}{60} \sin^{2}(2\omega ) \biggr) . \end{aligned}$$

Therefore, by the Riesz lemma, we have

$$\begin{aligned} H_{3}(z) ={}&{-}0.000015-0.00048z-0.00292z^{2}-0.010533z^{3}-0.028055z^{4} \\ &{}-0.036648z^{5}+0.058293z^{6}+ 0.26171z^{7}+0.472698z^{8}+0.285933z ^{9}. \end{aligned}$$

By (1.1) and (1.2) we obtain the two-scale relation

$$\begin{aligned} \phi_{3}(x) ={}&{-}0.00003\phi_{3}(2x)-0.00096 \phi_{3}(2x-1) \\ &{}-0.00584\phi _{3}(2x-2)-0.021066 \phi_{3}(2x-3) \\ &{}-0.05611\phi_{3}(2x-4)-0.073296\phi_{3}(2x-5) \\ &{}+0.116586 \phi_{3}(2x-6)+0.52342 \phi_{3}(2x-7) \\ &{}+0.945396\phi_{3}(2x-8)+0.571866\phi_{3}(2x-9), \end{aligned}$$

and the corresponding wavelet

$$\begin{aligned} \psi_{3}(x) ={}&0.571866\phi_{3}(2x+8)-0.945396 \phi_{3}(2x+7) \\ &{}+0.52342\phi _{3}(2x+6)-0.116586 \phi_{3}(2x+5) \\ &{}-0.073296\phi_{3}(2x+4)+0.05611\phi_{3}(2x+3) \\ &{}-0.021066 \phi_{3}(2x+2)+0.00584 \phi_{3}(2x+1) \\ &{}-0.00096\phi_{3}(2x)+0.00003\phi_{3}(2x-1). \end{aligned}$$

**Figure 1:**
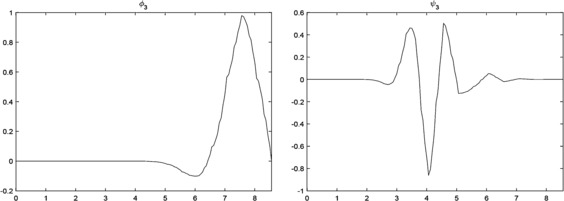
$\pmb{\phi_{3}(x)}$ **and** $\pmb{\psi_{3}(x)}$ **from Example** **3.1** **.**

In Figure 1, we show the graphs of $\phi_{3}(x)$ and $\psi_{3}(x)$, respectively.

### Example 3.2

Consider $m_{1}=2$, $m_{2}=3$, $a_{1}= \frac{1}{4}$ and $a_{2}=\frac{3}{4}$, then by Corollary 2.3, we have

$$\begin{aligned} \bigl\vert h(\omega )\bigr\vert ^{2}={}&\frac{1}{4} \biggl( 1-\frac{13}{15} \sin^{2}\omega - \frac{1}{30} \sin^{2}(2\omega ) \biggr) \biggl( 1-\frac{2}{3} \sin^{2}\frac{ \omega }{2} \biggr) \cos^{4}\frac{\omega }{2} \\ &{}+\frac{3}{4} \biggl( 1-\frac{2}{3}\sin^{2}\omega \biggr) \biggl( 1- \frac{13}{15}\sin^{2}\frac{\omega }{2}- \frac{1}{30}\sin^{2}\omega \biggr) \cos^{6} \frac{\omega }{2} \\ &{}+\frac{1}{2} \biggl( \frac{23}{15}\sin^{2}\omega + \frac{1}{30}\sin^{2}(2 \omega )-\frac{26}{45} \sin^{4}\omega -\frac{2}{90}\sin^{2}\omega \sin ^{2}(2\omega ) \biggr) \\ &{}\times \biggl( \biggl(1-\frac{2}{3}\sin^{2} \frac{\omega }{2} \biggr)\cos^{4}\frac{ \omega }{2}+\frac{1}{3} \sin^{2} \omega \\ &{} + \biggl(1-\frac{13}{15}\sin^{2}\frac{ \omega }{2}- \frac{1}{30}\sin^{2}\omega \biggr)\cos^{6} \frac{\omega }{2}+ \frac{13}{30}\sin^{2}\omega +\frac{1}{60} \sin^{2}(2\omega ) \biggr) . \end{aligned}$$

**Figure 2:**
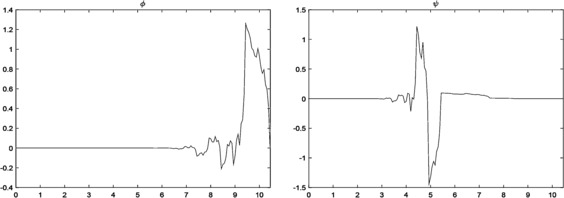
$\pmb{\phi (x)}$ **and** $\pmb{\psi (x)}$ **from Example** **3.2** **.**

Similar to the discussion of Example 3.1, we obtain $h(z)$, $\phi (x)$ and $\psi (x)$

$$\begin{aligned}& \begin{aligned} h(z) ={}&{-}0.0000003+0.0000003z-0.0000767z^{2} \\ &{}+0.0001077z^{3}-0.0029515z ^{4}+0.0041275z^{5} \\ &{}-0.0291355z^{6}+0.0401541z^{7}-0.0395286z^{8} \\ &{}+0.0473525z^{9}+0.5666672z ^{10}+0.4032326z^{11}, \end{aligned} \\& \begin{aligned} \phi (x) ={}&{-}0.0000006\phi (2x)+0.0000006\phi (2x-1) \\ &{}-0.0001534\phi (2x-2)+0.0002154 \phi (2x-3) \\ &{}-0.005903\phi (2x-4)+0.008255\phi (2x-5) \\ &{}-0.058271\phi (2x-6)+0.0803082 \phi (2x-7) \\ &{}-0.0790572\phi (2x-8)+0.094705\phi (2x-9) \\ &{}+1.1333344\phi (2x-10)+0.8064652 \phi (2x-11) \end{aligned} \end{aligned}$$

and

$$\begin{aligned} \psi (x) ={}&0.8064652\phi (2x+10)-1.1333344\phi (2x+9) \\ &{}+0.094705\phi (2x+8)+0.0790572 \phi (2x+7) \\ &+0.0803082\phi (2x+6)+0.058271\phi (2x+5) \\ &{}+0.008255\phi (2x+4)+0.005903 \phi (2x+3) \\ &+0.0002154\phi (2x+2)+0.0001534\phi (2x+1) \\ &{}+0.0000006\phi (2x)+0.0000006 \phi (2x-1), \end{aligned}$$

respectively (see Figure 2).

### Example 3.3

Let $a=6$, then, by Corollary 2.4, $b=-1$, $c=0$, $d=-5$, $e=0$. By (2.16), we have

$$\begin{aligned} \bigl\vert P(\omega )\bigr\vert ^{2}= \bigl(6-\sin^{2} \omega \bigr) \biggl( 1- \frac{2}{3}\sin^{2}\frac{ \omega }{2} \biggr) \cos^{4} \frac{\omega }{2}-5 \biggl( 1-\frac{13}{15}\sin ^{2} \frac{\omega }{2}-\frac{1}{30}\sin^{2}\omega \biggr) \cos^{6}\frac{ \omega }{2}. \end{aligned}$$

Now from the Riesz lemma, we have

$$\begin{aligned} P(z) &=0.00595-0.01085z-0.09675z^{2}+0.18428z^{3}+0.58577z^{4}+0.32154z ^{5}. \end{aligned}$$

**Figure 3:**
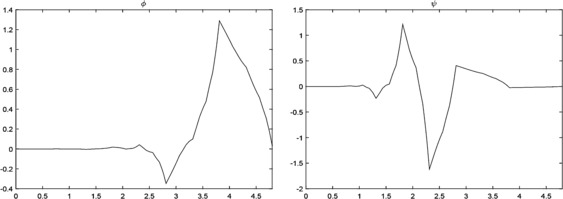
$\pmb{\phi (x)}$ **and** $\pmb{\psi (x)}$ **from Example** **3.3** **.**

Then we obtain from (1.1) and (1.2) the two-scale relation

$$\begin{aligned} \phi (x) ={}&0.0119\phi (2x)-0.0217\phi (2x-1)-0.1935\phi (2x-2)+0.36856 \phi (2x-3) \\ &{}+1.17154\phi (2x-4)+0.64308\phi (2x-5), \end{aligned}$$

and the corresponding wavelet $\psi (x)$ (see Figure 3)

$$\begin{aligned} \psi (x) ={}&0.64308\phi (2x+4)-1.17154\phi (2x+3)+0.36856\phi (2x+2)+0.1935 \phi (2x+1) \\ &{}-0.0217\phi (2x)-0.0119\phi (2x-1). \end{aligned}$$

## Conclusion

A simple and flexible method for constructing compactly supported orthogonal scaling functions is presented in this paper. Using this method, we can construct orthonormal compactly supported scaling functions from *B*-splines. Note that the change of $\lambda_{i}$ ($i=1, \ldots, N$) can cause the change of the scaling functions corresponding to two-scale symbol $H(\omega )$ in (2.8). Therefore we can provide the user with different scaling functions with the same compact support. Similarly, then we can obtain different compactly supported scaling functions by changing the parameters in (2.10).
